# Dataset of *in-situ* coaxial monitoring and print’s cross-section images by Direct Energy Deposition fabrication

**DOI:** 10.1038/s41597-023-02672-4

**Published:** 2023-11-07

**Authors:** Javid Akhavan, Jiaqi Lyu, Youmna Mahmoud, Ke Xu, Chaitanya Krishna Prasad Vallabh, Souran Manoochehri

**Affiliations:** Stevens Institute of Technology, Hoboken, USA

**Keywords:** Databases, Mechanical engineering

## Abstract

Coaxial monitoring of the Direct Energy Deposition (DED) machines enables a real-time material deposition study. Coaxial-images contain substantial melt-pool information and incorporate situational information including the sparks’ intensity, numbers, etc. Recent studies have shown that melt-pool observations correlate directly with machine parameters and artifact properties. Therefore, the melt-pool information not only can assist in measuring the machine’s working condition and determining machine operation parameters’ reliability but also facilitates the deposition characteristics studies like print’s regime and dimensions. This information is gathered during the fabrication and can be expanded to perform various process studies and fault registration. This paper utilizes the Optomec DED machine to fabricate single-track prints with multiple process parameters, while a coaxial camera records the deposition. Each deposited track is then cut perpendicular to the print’s direction to facilitate process parameters correlation study with actual geometrical deposition measured using a microscope. The coaxial images taken during fabrication, along with their process parameters, cross-cut measurements, and a developed image-processing toolbox, are presented alongside this paper to empower future research directions.

## Background & Summary

Metal additive manufacturing, also known as 3D printing, is a process that involves building up layers of metal material to create a physical object. It offers several benefits over traditional manufacturing methods, including the ability to produce complex shapes and the potential for reduced material waste. However, the adoption of metal additive manufacturing in the industry has been limited due to the lack of reliable process-structure-property relationships, which makes it difficult to predict how a part will perform based on the manufacturing process used to produce the part^1^.

One specific metal additive manufacturing process is laser-directed energy deposition (L-DED), which involves using a laser beam to melt and deposit metal material in a controlled manner^2^. While L-DED has gained attention in recent years, it is still not as reliable and repeatable as traditional manufacturing tools. This could hinder its industry adoption since manufacturers must consistently produce high-precision parts to meet customer demands. Due to the intricate physics of the laser-material interaction and the cyclic thermal loading that takes place during metal additive manufacturing, various issues may arise, including residual stresses, porosity, inferior dimensional accuracy, and impaired mechanical properties^3–5^. These problems may be caused by a variety of factors, including the process parameters used during the manufacturing process and the material being employed. Residual stresses, for instance, may be caused by the difference in thermal expansion between the laser-heated material and the cooler surrounding material, while porosity may be caused by a lack of fusion between the layers of material. Poor dimensional accuracy may be caused by a variety of factors, including temperature variations, material shrinkage, and laser beam focus. Impaired mechanical properties may be caused by the formation of defects in the material structure during the manufacturing process. To optimize the process parameters and produce high-quality parts, it is necessary to fully grasp the process-structure-property relationship (PSP), which refers to how the manufacturing process affects the structure and properties of the resulting part^4^.

The process of fabricating parts using laser-directed energy deposition (L-DED) is governed by numerous process parameters, including laser power, laser scan speed, and powder feed rate. To study the effects of these process parameters on the microstructure and physical properties of samples and determine optimal conditions for manufacturing larger builds, single-track deposits/prints are often utilized in metal additive manufacturing due to their simplicity^6–8^. By fully understanding the effects of these process parameters on the resulting parts, researchers and manufacturers can optimize the L-DED process to produce high-quality parts consistently.

By examining data obtained from online monitoring sensors, researchers have discovered correlations between various process parameters, including laser power, scanning speed, powder feed rate, and melt-pool dimensions through the application of observations and statistical methods. This information can be utilized to optimize the L-DED process and enhance the reliability and repeatability of metal additive manufacturing.

In the field of metal additive manufacturing, researchers have studied the effects of various process parameters on the melt-pool width, temperature, and track height. For example, Bi *et al*. found that laser power had a minimal effect on track height but a strong influence on width^9^, while Ocylok *et al*. determined that laser power had the strongest correlation to the melt-pool size and that powder feed rate had a minimal impact on melt-pool size^10^.

Melt-pool data has been increasingly used for defect detection and prediction, leading researchers to combine microstructure analysis and melt-pool dimension measurement with online monitoring. High-speed cameras^11,12^ and Inline coherent imaging^13^ are among the recent examples of correlating melt-pool observations to balling, keyhole porosity, and print continuity information achieved from post-processing microstructure analysis. To detect errors, Clijsters *et al*. employed a thermal CMOS camera and a photodiode to capture near-infrared (NIR) melt-pool areas and intensities^14^.

A variety of non-contact measurement methods have become increasingly popular among researchers for capturing the thermal history and melt-pool temperatures of L-DED and other metal AM processes. These methods include optical tomography, high-speed cameras, photo-acoustic imaging, and infrared pyrometers^12–19^ Part quality could be determined by the temperature distribution in the melt-pool^18^. As part of the thermographic inspection, Marshall *et al*. utilized a dual-wavelength pyrometer and an in-chamber infrared camera to observe the melt-pool temperature and the part’s temperature history^15^.

Given the significance of having real time observation of DED additive manufacturing process, in this article, a dataset^20^ of various coaxial observations from experiments with different process parameters during the DED fabrication process described in the methodology paper^21^ is structured. Alongside the coaxial camera data, each printed track was cut to achieve cross-sectional images representing how the resulting print was for various process parameters. This dataset can be utilized to further study the melt pool dynamics and parameter transitions.

## Methods

### Data collection

In this work, 328 single scan track (SST) samples were printed, with varying laser power, scanning speed, and powder feed rate. The goal was to understand how these process parameters influence the morphologies of SSTs. The laser power, print speed, and powder feed rate were tested within the ranges of 200–500 W, 10–1000 mm/s, and 0.25–15 g/min, respectively, as shown in Table 1. To ensure stable and defect-free prints, the process parameter combinations were carefully chosen based on empirical selection, keeping the linear energy density between 40 and 100 J/mm.

**Table 1:** Process parameter values used in the experiments.

Process Parameter	Test Range
Laser Power (W)	200–500
Print Speed (mm/min)	10–1000
Powder feed rate (g/min)	0.25–15
Argon shielding gas flow (L/min)	30
Argon carrier gas flow (L/min)	4

The SSTs’ fabrication was carried out using an Optomec LENS MTS 500 printer. This advanced printer is equipped with a two-powder feeder hopper, a CNC control system, and a deposition head capable of reaching a maximum laser power of 500 W, operating at a center wavelength of 1070 nm.

**Fig. 1:**
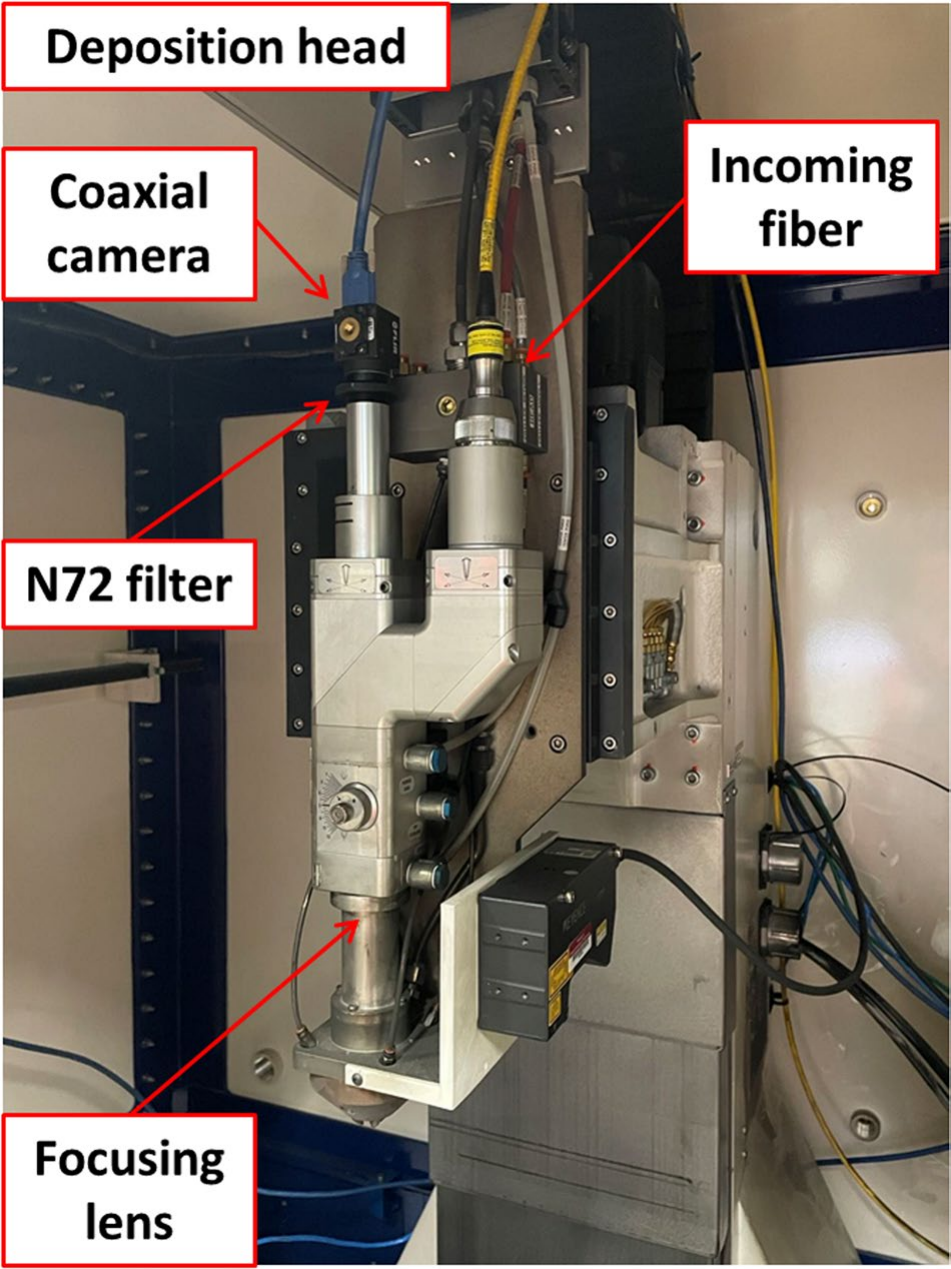
The experiment setup for the Coaxial camera.

The fabrication process takes place in a fully inert controlled chamber, where a 76.2 mm × 101.6 mm × 8 mm SS 316 L substrate is used. The process involves continuously blowing SS316 powder through four lateral nozzles while simultaneously ejecting argon gas to precisely direct the extrusion to the focal point of the high-powered laser. This ensures the shielding of the deposited metal from contamination and oxidation. This DED experimental setup is shown in Fig. 1. For the sample fabrication, Stainless Steel 316 L powder from CARPENTER ADDITIVE, with a diameter range of 45–106 µm, was utilized. The chemical composition of the powder is presented in Table 2.

**Table 2:** Metal powder properties.

Element	Mo	N	C	Cr	Mn	Ni	Si	P	S
wt%	2	0.1	0.03	16–18	2	10–14	1	0.045	0.03

After printing the single scan track samples are printed, they undergo a series of processing steps to prepare them for microstructure analysis. In this step, the sectioning machine, mounting machine, and polishing machine from Allied High-Tech Products, Inc., specifically models TechCut 5x™, TechPress 3™, and MetPrep 3™, respectively, are employed.

**Fig. 2:**
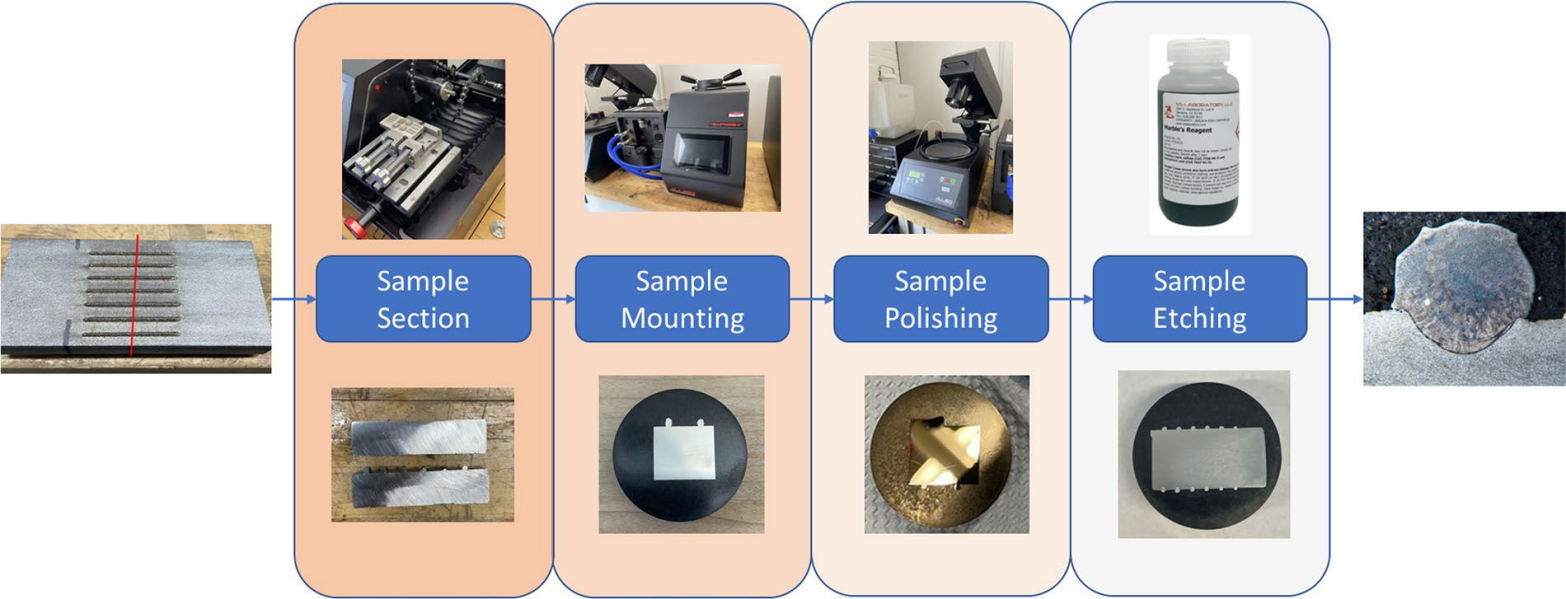
Illustration of sample processing steps.

First, the samples are sectioned perpendicularly to the scan direction by an aluminum oxide blade operating at 3000 rpm. Sectioned samples are then rinsed with ethyl alcohol, dried with compressed air, and then mounted into a 30 mm cylinder using graphite-based conductive powder. This provides a good edge retention and hardness for SEM usage. The molds with the SS316L cross-sections of interest are then polished with 1200 Grit Silicone Carbide paper and etched using Marble’s reagent etching solution for 45 s. This ensures a better visualization of the cross-section’s microstructure and macrostructure when inspected. This process is summarized in Fig. 2.

**Fig. 3:**
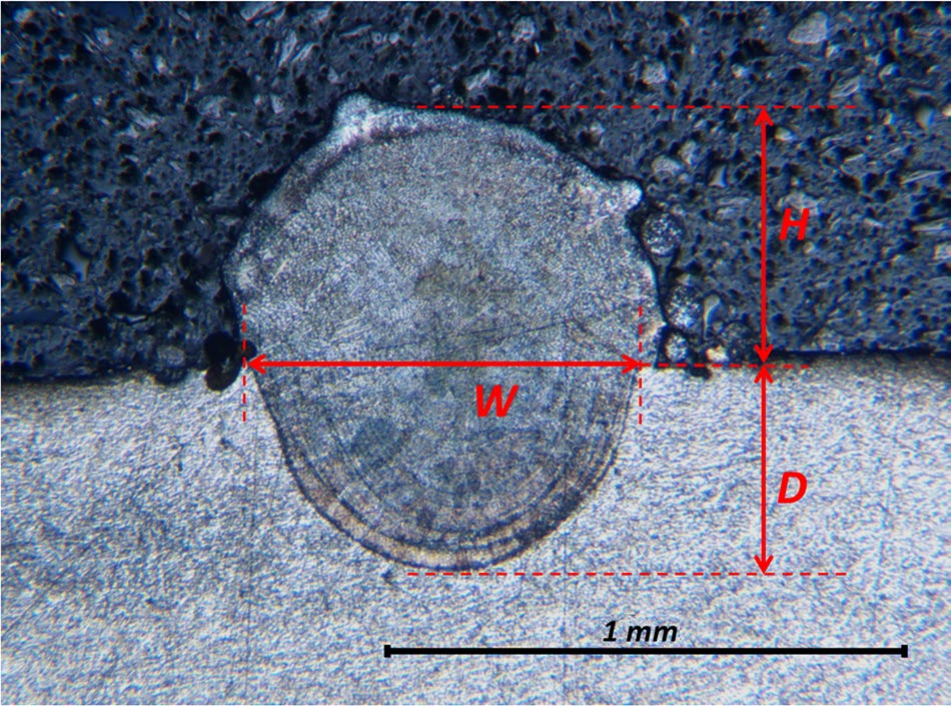
Definition of D, W, and H for the cross-section data.

After sectioning and etching, the samples’ cross-sections are examined using an optical Olympus BH-2 Microscope, with a magnification of 5X. Using the microscope, a picture as the one shown in Fig. 3 is taken. Each picture is then labeled with a unique name tag that is pre-defined for every cross-section, encompassing the part number, track number, and the assigned letter for identification purposes. To measure the three geometrical attributes of interest, namely, the track width (W), depth (D), and height (H), the pictures are loaded onto the suggested application, AM-scope, where they are measured in pixels and stored in an Excel file. This process results in an Excel file with the section’s number, process parameters, and measured dimensions.

### Data pre-processing

**Fig. 4:**
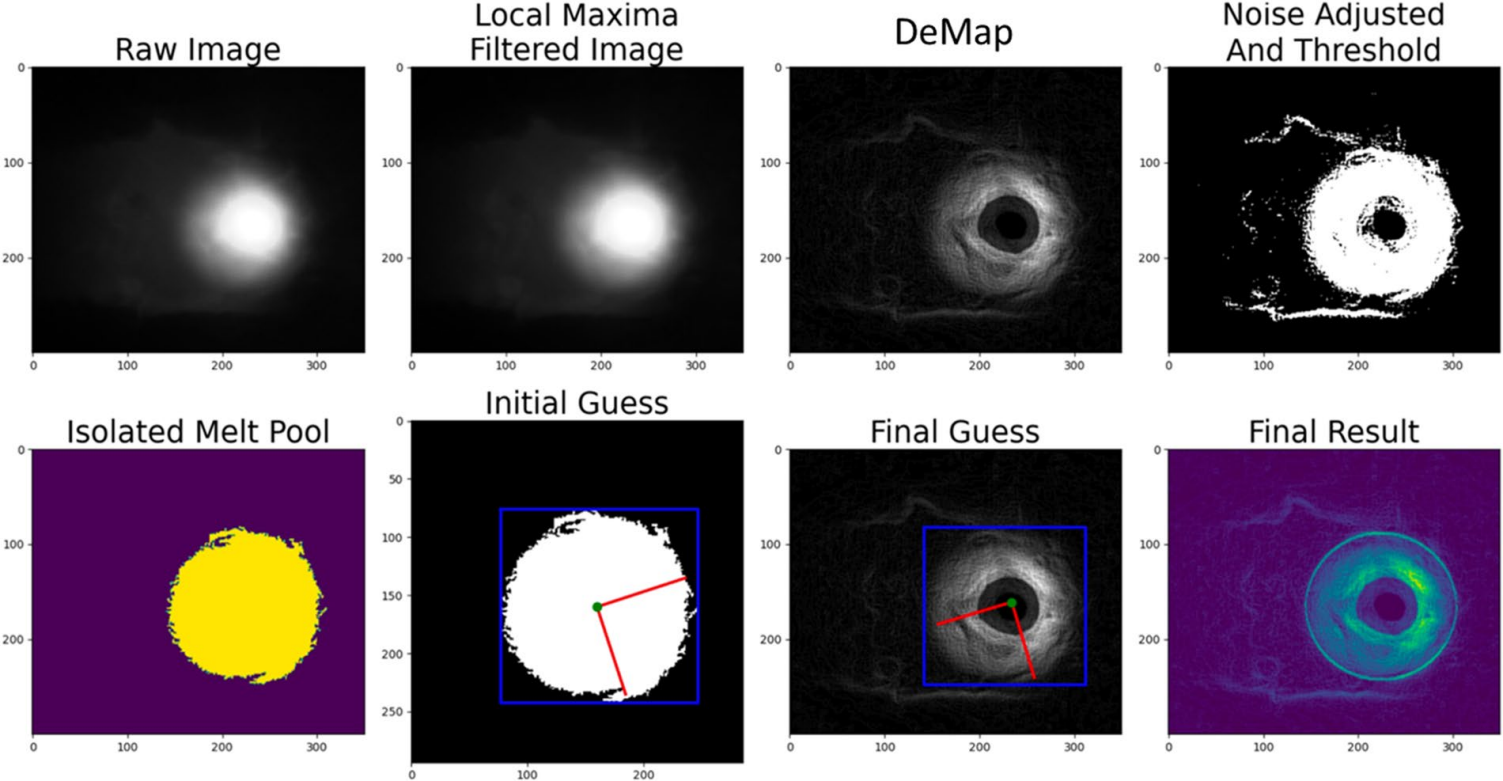
Resulting images from each step taken by the pre-processing algorithm.

The acquired denoised data from the coaxial camera, during the DED fabrication process, shows both the melt-pool geometries and signatures, along with sparks, residual powders, and random reflections from the print bed. However, because this study is solely focused on the melt-pool features, it is necessary to distinguish its signatures and eliminate the surrounding information and noise within the images. For example, a multi-frame denoise is used to remove the spark path. For every two adjacent frames, the pixels at the same location of every two adjacent frames are compared. The minimum value is picked to be the final denoise image. A configurable noise detection and elimination program is also developed. This program allows full control over all the steps taken for image processing by getting all the required assumptions as inputs. Figure 4 depicts the denoising algorithm flowchart. Starting from the top, once images are located and loaded, a local maxima filter with an adjustable filter size is applied to achieve the Deviation Map (DeMap). Once the effect of the DeMap is applied, a tunable hard thresholding is applied to achieve a binary image. The resulting binary image might consist of islands due to a strong presence of sparks, noise, or reflection. Therefore, by region growing method, the largest continuous region is distinguished and kept as the melt-pool.

### Deviation Map (DeMap)

The DeMap represents regions containing an intensity gradient that is correlated with the region’s temperature map. Since the DeMap and how it is applied for denoising can result in various meaningful information, multiple approaches are implemented in the image processing code.

The DeMap alone can provide meaningful information to pinpoint regions with fluctuating intensity and temperature. When analyzing the DeMap, it is possible to separate the background from the melt-pool core to keep the temperature-varying regions, such as solidifying regions. The solidifying section can be further used to analyze the time of glass transition, rate of cooling, and many other aspects that rely on observations linked to after-melt events.

On the other hand, the addition or subtraction of the DeMap from the raw image can result in the segregation of various melt-pool aspects. Subtraction of the DeMap from the raw data helps eliminate fluctuating temperature signatures and noise, which allows localization of the laser beam core and stable melt-pool core. Using the acquired melt-pool core readings, one can determine not only laser power and its penetration into the build plate but also the printing regime and quality of the deposition. On the other hand, the addition of the DeMap to raw data can illustrate the varying temperature maps effect. This in turn generates an outer-bound melt-pool geometries analysis. Despite indicating unwanted noise, this method also assists in the melt-pool’s outer boundary detection. A melt-pool’s outer boundary contributes significantly to the analysis of the print’s resulting track geometry, and the detection of sparks, and unwanted anomalies.

Therefore, the program is designed to incorporate all three methods mentioned above with configurable parameter input. To make the code more generalizable, all the assumptions and constants needed for image processing and information extraction are parameterized. Therefore, depending on the task, even with a different setup, camera, and input properties, hyperparameter analysis, and tuning have been made customizable and easily accessible.

### Ellipse fitting

**Fig. 5:**
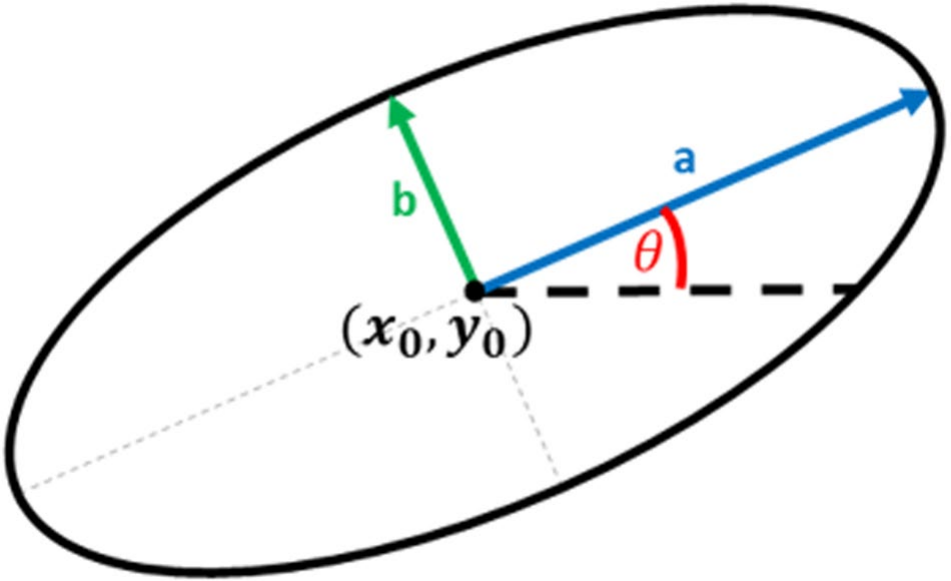
Illustration of the ellipse model used for melt-pool representation. X0 and Y0 are the ellipse center coordinates. A and B are the longest and the shortest ellipse diagonal respectively. *θ* is the ellipse’s tilt.

The size of the melt-pool and various geometrical information about melt-pool can be further used to study the undergoing print and fabrication quality and in general print’s dynamic. Therefore, as suggested by literature^22,23^, the closest geometrical form to represent a melt-pool is using an ellipse. To that end, it is necessary to find the best ellipse that fits the melt-pool and achieve the key parameters. Each ellipse can be defined by five hyperparameters as longest (a) and shortest (b) diagonal, X and Y coordinates of the center, and orientation (θ). The ellipse model is depicted in Fig. 5. Two approaches are implemented for the parameter estimation, naive and grid search which will be discussed in detail.

### Naïve approach

In the naïve step, the normalized second central moments of the region are calculated. Then, based on the calculated moments, the ellipse with similar normalized second central moments is chosen as an approximated geometry. Therefore, with a relatively fast calculation, an acceptable ellipse is achieved. To step further and fine-tune the ellipse to fit best into the melt-pool, a grid search algorithm is implemented.

### Grid search (GS) approach

The grid search approach, although requires a significant processing time, is guaranteed to achieve the local minima and converge to the best available solution. To accelerate the search and narrow down the search domain, for each parameter, the rough estimations achieved by the normalized second central moments approach are used. Therefore, the GS iterates only over a small range of possible candidates. Also, to fasten the search, for each parameter, a step size is defined to skip very close iterations. The scope of the search for each parameter and the corresponding step size is tunable bypassing the upper, and lower hand flexibility, and step size for each parameter to the search algorithm.

Once the search scopes are defined, for each set of possible parameters, starting from the largest diagonal sets, an ellipse mask is generated. Then, by comparing the generated mask and the input image, the cross-correlation of the two is calculated. The largest diagonal which achieves a +97% cross-correlation value would be picked as the best fit. The GS algorithm pseudo-code is illustrated in Table 3.

**Table 3:** Grid Search algorithm flowchart to find the best elliptical fit.

### Dataset creation

**Fig. 6:**
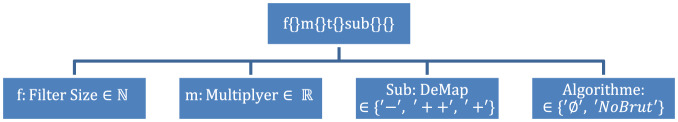
Dataset generation naming policy.

Once the evaluation of the geometrical parameters is done, the calculated values are then stored as a database file with the appropriate name tag based on the image processing hyper-parameters. The format of the name tag is made by hyper-parameter abbreviation with the corresponding value, as shown in the Fig. 6.

## Data Records

**Fig. 7:**
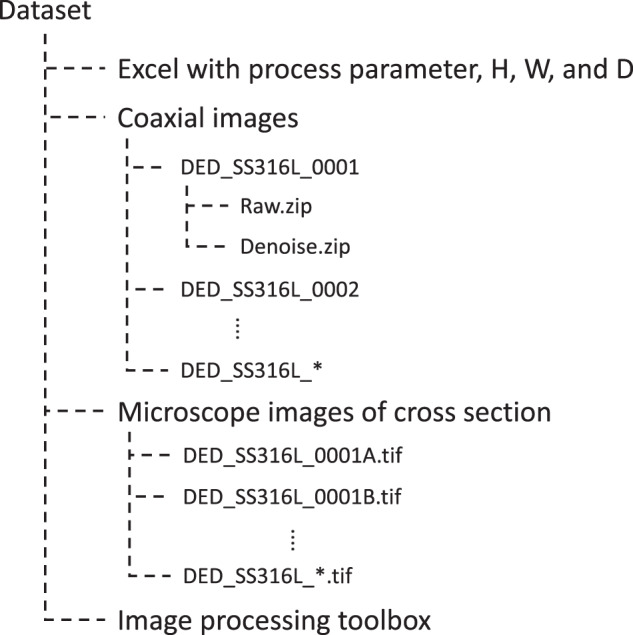
Dataset file structure tree.

The structure of the dataset folder, deposited in Scientific Data Bank, a general-purpose data repository^20^ is shown in Fig. 7. The database consists of four main sections, a main Excel file, coaxial images, crosscut images, and an image processing toolbox.

**Fig. 8:**
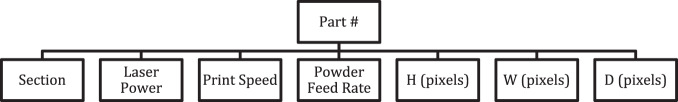
Excel file structure.

First is an Excel file in which all the parameters for each SST are stored. Each data sample in the Excel file includes printing process parameters, and the cross-section measurements (height, width, and depth). Figure 8 depicts the Excel file headers.

Second, In the coaxial images folder, under each SST sample name tag, the raw images and corresponding denoise images are included as a zip file. The coaxial images follow a name code as prim *_**.npy. which * is the placeholder for the image’s index during the recording and ** is the machine time in seconds which is obtained by the python library time. time. The format of the raw images is.npy. It can be accessed by using the Python library Numpy load function. The samples raw images from tag number 0000 to 0056 are 8-bit RGB images with shapes as (800, 800, 3). Starting from sample number 0057, the raw images are saved as 16-bit grayscale images with shapes as (800, 800, 1). The denoising methods used for this database will be discussed in the code availability section. The denoised images are saved as 8-bit grayscale JPEG images.

Third, crosscut images obtained by expert researchers are stored. These images were measured in height/width/depth bases. The format of crosscut images is *.tiff which also includes measurement information by AM-scope application.

Last, is the image processing toolbox folder which includes all the functions and codes developed for processing this database and generating outputs.

## Technical Validation

As described in the previous sections, the experiments are conducted on the DED system with different process parameters. In this section, dataset validation for process parameters, melt-pool coaxial images, and single-track cross-section are performed.

### Validation of process parameters

**Fig. 9:**
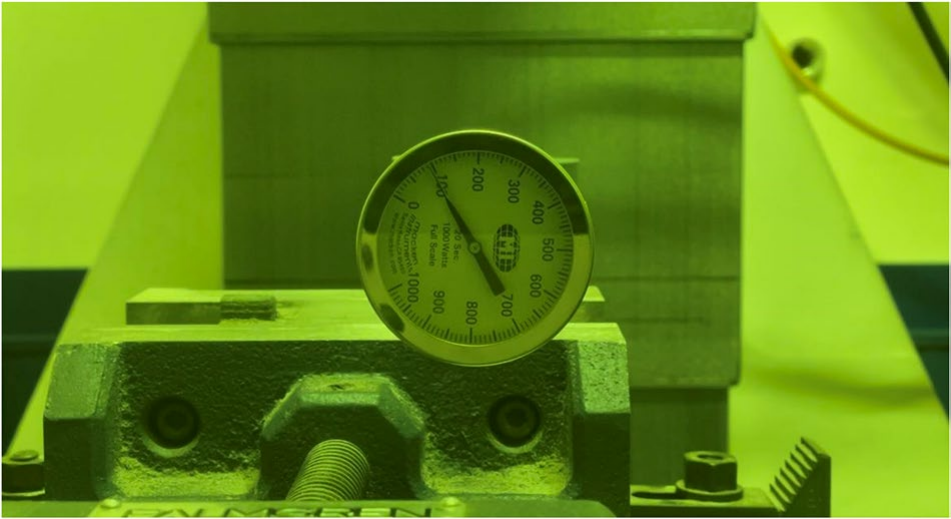
Laser power validation.

In the experiments, three process parameters, namely laser power, scanning speed, and powder feed rate, are changed in the experiments and the rest are kept constant, For the laser power, the Macken Instrument analog thermopile laser power meter, 100–500 W YAG&CO2 is used to calibrate the laser power, as shown in Fig. 9. The laser power is measured with the setting of 100, 200, 300, 400, and 500 W(Max). Based on the validation measurement, the tolerance of laser power of DED is confirmed within ±5%. The scanning speed of DED is validated by timing the movement of 100 mm. The scanning speed tolerance is within ±1%.

The unit of powder feed rate is RPM in this dataset. The relation to the gram/min is shown in Table 4.

**Table 4:** Powder feed rate from RPM to g/min.

RPM	gram/min (±0.2)
0.5	0.7
1	1.7
2	2.7
3	4
4	5.7
5	6.6
6	7.9
7	9.2
8	10.5
10	14.1
12	17
14	18.9
15	20.1

### Validation of coaxial images

**Fig. 10:**
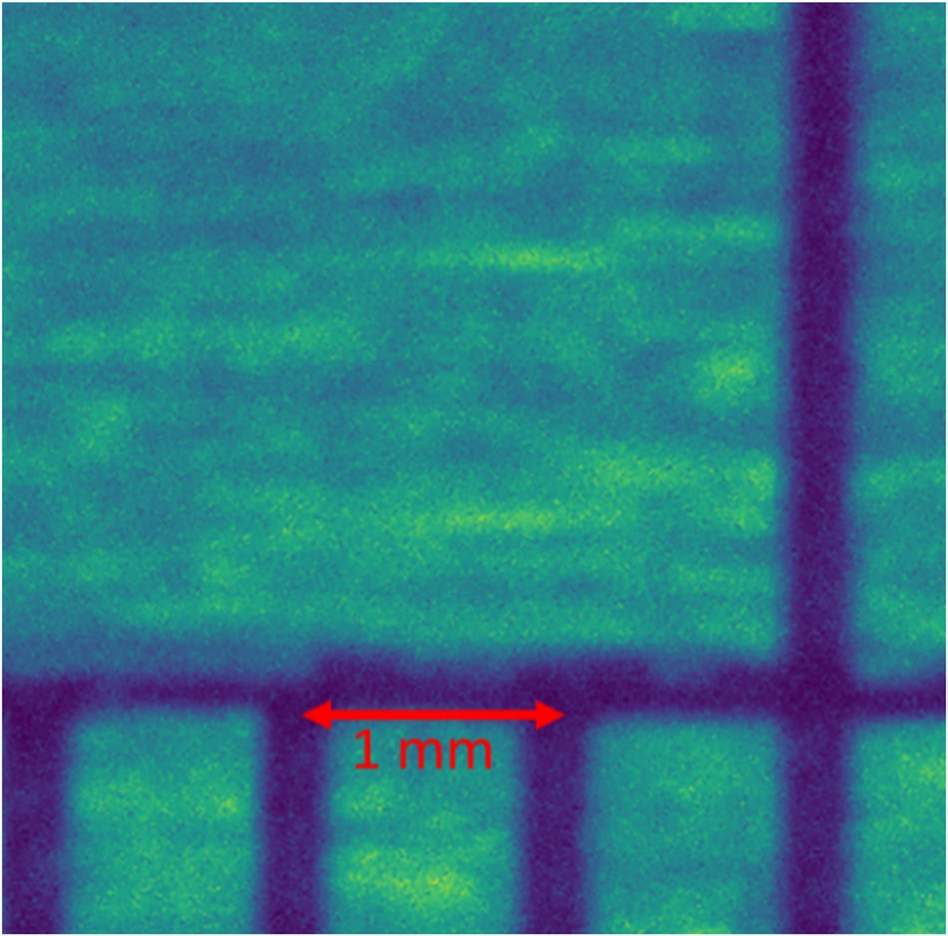
Coaxial camera calibration scale.

The coaxial camera for this study is mounted on the feeder’s head directly above the laser hit point. To ensure the reliability and repeatability of the experiment, camera focus, and calibration are necessary. Therefore, to calibrate the camera, first, the machine with the disarmed laser is moved above the print bed at the same height as a normal print would take place. Then, by manually adjusting the camera lens, the focal point is moved to achieve the clearest output. Thereafter, to calibrate the camera, a calibration sheet is placed underneath the feeder and a few pictures are taken, as shown in Fig. 10. For the coaxial camera, we have verified our calibration results by comparing our actual measurements against the sensor resolution reported. We used rulers in different directions to obtain observable resolution as reported our pixel-to-real scale measurement shows 1 mm = 220 pixels. This number matches with the sensor resolution of 4.5 µm/pixel reported by the manufacturer (FLIR). Using the pixel conversion value, it is possible to convert the observed deposited track width with the actual deposition to make sure the transformation and observations are in line.

### Validation of single-track cross-section images

**Fig. 11:**
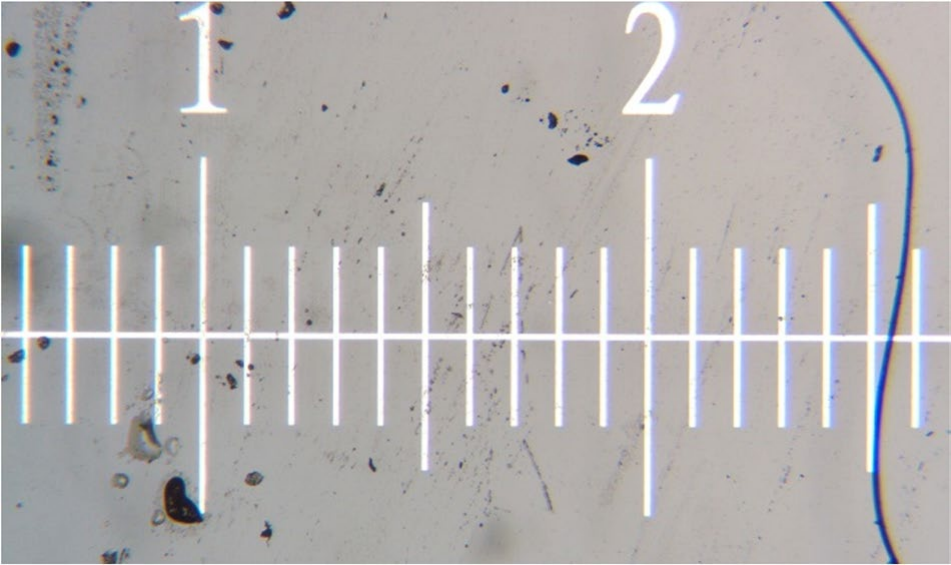
Microscope calibration ruler.

Once the fabrication is done, using a destructive approach, every single track is cut perpendicular to the print direction to obtain a cross-sectional observation. These observations are crucial in understanding the actual print outcome and quality. To make sure the observations are reliable and accurate, first, a name tag system was implemented to track each specimen and corresponding information. Then the Olympus BH-2 Microscope is calibrated by the manufacturer’s recommendation procedure. A calibration ruler is used to find the real dimension of each pixel under 5X magnification, as shown in Fig. 11. The pixel-to-real scale for the microscope is 1 mm = 1202 pixels. Then the height, depth, and width of the melt-pool are measured by expert researchers, as described in previous sections.

## Usage Notes

For this study, each track might contain various parameters and printing conditions. At the transition point, where the machine is adjusting to the new set of parameters, for a brief time, the process undergoes a transition. This could be acceleration to adjust the speed or a lag in feed rate due to the feeder pump inertia. This transition was captured by the coaxial camera and can alone be a great opportunity to get deeper and analyze the effect. However, to correlate the process parameters and coaxial observations to the print’s regime and SST’s geometry, it is necessary to observe the steady-state fabrication. Therefore, to make sure the observed data is free of any transition, the user can drop the first and last 15% of the data corresponding to each track.

It is worth mentioning the dataset contains experiments with various fabrication speeds. Therefore, the faster the print speed is, the shorter the fabrication time gets. With a fixed frame rate per second data acquisition, the number of images per experiment varies. To address this unbalanced number of images available per experiment, it would be a reasonable attempt to randomly sample a fixed number of images from each folder to make sure each experiment will have the same number of data points.

Although the co-axial images are not timestamped, it is possible to register them in the time and tool path coordinates using the process parameters. As all the images are taken with the same frame rate, known printing speed for each track, and known track length it would be possible to calculate each frame’s time and location to create a spatiotemporal link .

**Table 5:** Image processing function input description.

Keyword	description	Default Value	Data type
Inp_image	Input image for analysis	[]	2D-array of type float32
save_figs	Saving figure outputs as a separate file. 0: no figures will be saved separately 1: All the mid-process steps’ figures will be saved separately	0	1 × 1, Boolean
filter_size	Definition for the filter size used in the image processing to calculate the DeMap.	10	1 × 1, integer
multiplier	Definition for the multiplication value used in the image processing algorithm to apply DeMap.	5	1 × 1, Float32
threshold	Definition for the threshold value used in the image processing steps. Depending on the algorithm selected, appropriate values vary. For cases of addition higher values of threshold are suggested while in the case of DeMap alone, very small values are useful.	0.3	1 × 1, Float32 ∈[0, 1]
raw_out	The raw output flag is used to determine if the user needs the final image to be built from the processed data, or the raw input image. 0: Processed and binary image is cropped and returned 1: Raw image is cropped and returned	0	1 × 1, Boolean
sample_plot	Sample plot flag is used to activate mid-stage steps illustrations. After each step, the data is stored and plotted for the user to show the effect of the steps taken on the input image. 0: Turning off the mid-stage plots 1: Turning on the mid-stage prints (It is worth mentioning that this feature is developed for debugging and fine-tuning purposes and for the actual run and database processing this must be turned off to allow multicore processing capabilities)	0	1 × 1, Boolean
substract_flag	The subtraction flag is used to pick the algorithm used in image processing. There are 3 available methods: 0: Using an addition formula to apply the DeMap over the input image. 1: Using a subtraction formula to apply the DeMap over the input image. 2: Using the DeMap as the base	0	1 × 1, Float32 ∈{0, 1, 2}
file_name	The file name value is used to name the output files if there is one requested.	‘’	String Array
folder	The folder address is used to clarify the location in which the output file if there is one requested be saved. In the specified location, a folder by the name: resulting_images is created to store the resulting images	‘’	String Array
extend_const	An extension constant is used as to how big the resulting image dimensions will be. Once the melt-pool core coordinates are measured, an output image with pixel dimensions, with double this value centered at melt-pool core coordinates is generated.	128	1 × 1, Integer
skip_brut	Skip brute force flag is used to determine which estimation algorithm to use for the ellipse approximation. 0: Using the brute force method after a fast estimation model to fine-tune the parameters found 1: Using the faster model based on normalized second central moments to estimate the ellipse parameters	0	1 × 1, Float32 ∈{0, 1}
brut_opts	Placeholder used to pass the brute force algorithm hyperparameters. [upper_range_x, lower_range_x, upper_range_y, lower_range_y, upper_range_a, lower_range_a, upper_range_b, lower_range_b, tetha_step, step_x, step_y, step_a, step_b]	[]	1 × 13, Float32

**Table 6:** Image processing function output description.

Place holder	Description
0	Cropped binary image centered at the melt-pool
1	Full-size resulting image after image processing
2	Melt-pool’s core X coordinate, uncropped image coordinate
3	Melt-pool’s core Y coordinate, uncropped image coordinate
4	Melt-pool’s core’s largest diagonal length
5	Melt-pool’s core’s Shortest diagonal length
6	Extension value used for the output images
7	Cropped raw image centered at the melt-pool
8	Initial guess for the ellipse model. [cx_centered, cy_centered, radii, radii_s, orientation]
9	Success flag for ellipse detection. 0: ellipse was found successfully 1: no ellipse was found 3: No area was available for detection

## Data Availability

The source codes used for data generation and initial image processing for melt-pool detection and geometrical analysis are also available along with the data. These codes can serve as a useful tool for future integration. The descriptions of the inputs and outputs of the used image processing function are summarized in Tables 5, 6, respectively.
